# Frontline professionals’ use of and attitudes towards technology to support interventions for adolescents with depression symptoms: A mixed methods survey

**DOI:** 10.1177/13591045231212523

**Published:** 2023-11-06

**Authors:** Maria E Loades, Bethany Cliffe, Grace Perry

**Affiliations:** 1 Department of Psychology, 1555 University of Bath, UK; 2 Department of Psychology, University of Southampton, UK

**Keywords:** Attitudes, children, adolescents, digital mental health interventions, single session interventions, frontline professionals

## Abstract

Depression is common in adolescence, and subthreshold symptoms even more so. Untreated, it is disabling. Yet, upscaling traditional clinic-based provision would be prohibitively expensive. We aimed to investigate frontline, non-specialist professionals’ use of and attitudes towards technology to increase the availability of early help.

**Method:**

Cross-sectional survey of a convenience sample of professionals in the UK (*N* = 115, including low intensity practitioners, GPs, education staff, school nurses). The survey included rating scales and free text boxes. Quantitative data were analysed descriptively, and we used reflexive thematic analysis for the qualitative data.

**Results:**

Frontline professionals rate their technological competence as good and have favourable attitudes towards using technology to support adolescents with depression symptoms. They rated online resources as most useful with mild-moderate symptoms, compared to severe symptoms (*t*(110) = 14.54, *p* < .001, Cohen’s *d* = 1.49). Technology was viewed as important to bridge the needs-access gap and professionals were interested in learning about online SSIs due to usefulness (*r* = .32, *p* < .001).

**Conclusion:**

Technology, such as SSIs, are of interest to mental health professionals and may be useful for supporting adolescents with depression. Future research should explore the use of SSIs for treating adolescent depression.

## key practitioner message

• Digital technology offers an effective way to deliver evidence-based treatment at scale.
• Frontline, non-specialist professionals may often be the first point of contact for adolescents when they begin to struggle with their mental health, including with depression symptoms.
• Therefore, they may be well-placed to signpost adolescents to digital mental health interventions, including single session interventions (SSIs).
• We found that UK based frontline professionals have favourable attitudes towards using technology to support adolescents with depression symptoms, particularly for those with mild-moderate symptoms as compared to those with severe symptoms.
• Technology was viewed as important to bridge the needs-access gap.
• Professionals were interested in learning about online SSIs.

## Introduction

Depression becomes more common during adolescence (Lu, 2019; Rapee et al., 2019). By age 18, one in ten adolescents will have had depression (Solmi et al., 2021), characterised by sadness and/or irritability and lack of enjoyment/motivation (A.P.A., 2013), and one in three adolescents has elevated depression symptoms currently (Shorey et al., 2021). Adolescent depression has substantial short- and long-term consequences and costs, which prompt treatment can decrease (Goodyer et al., 2017). Yet, despite the burden of adolescent depression, we do not have the clinic-based resources to treat every adolescent with elevated depression symptoms and upscaling existing provision would be prohibitively expensive. Unsurprisingly, need far outweighs capacity, even in early help services, so adolescents must wait for help and this needs-access gap has increased further post-pandemic (Huang & Ougrin, 2021). Therefore, we need low cost, widely available, acceptable, and effective interventions to prevent adolescents’ depression symptoms from escalating.

Digital technology offers an effective way to deliver evidence-based treatment at scale (Taylor et al., 2020). Digital mental health interventions are effective (Grist et al., 2018; Lehtimaki et al., 2021; Vigerland et al., 2016) and can overcome many help-seeking barriers, including stigma and practicalities like travel and timings of appointments, with reduced costs to the healthcare system. However, uptake and engagement in using digital mental health interventions has been problematic; effective treatments like internet-based cognitive behaviour therapy (iCBT) suffer from significant drop out; for example, one third of anxious children and adolescents (*N* = 4425) who enrolled in an open access 10 session self-help iCBT programme only used one session, and less than a third of those who started the programme completed ≥3 sessions (March et al., 2018). Digital single session interventions (SSIs) intentionally designed as one-off therapeutic self-help interventions with no assumption of a return visit may therefore be a particularly scalable public health solution as an addition to current provision for adolescent depression symptoms (Loades & Ching, 2021; Loades & Schleider, 2023).

Most often, frontline education and healthcare professionals, such as General Practitioners (GPs), teachers, school nurses, and low-intensity (non-specialist) wellbeing practitioners (Education Mental Health Practitioners - EMHPs, Child Wellbeing Practitioners - CWPs) based in Mental Health Support Teams (MHSTs) in schools and in community settings, will be the first professional source from whom adolescents and their families will seek support. We therefore need to understand how these frontline professionals, who signpost adolescents to early help and/or act as gatekeepers to specialist services view digital mental health intervention options, including single session interventions, as their attitudes towards these options are likely to influence whether and how they offer them to adolescents.

Several prior studies have investigated child and adolescent mental health professional’s attitudes towards digital mental health interventions. In the UK, a survey of a convenience sample of 43 specialist cognitive behaviour therapists’ attitudes towards the use of computerised CBT (cCBT) with children and adolescents found cautious optimism, with particular relevance perceived to be for preventative and early intervention approaches (Stallard et al., 2010). A subsequent UK survey of 154 specialist Child and Adolescent Mental Health Service (CAMHS) professionals found that perceived benefits of technology to provide and support interventions included accessibility, convenience and appeal, although similar to the prior study, it was perceived to be more useful for prevention or psychoeducation (Cliffe et al., 2019). A Swedish study also surveyed 156 child and adolescent mental health professionals from 15 services, finding generally low knowledge of cCBT, and again, more optimism about its use for prevention and mild-moderate problems (Vigerland et al., 2014). However, these studies all focused on specialist staff to whom an adolescent would typically be referred, rather than frontline professionals working in community settings who might first encounter an adolescent who is struggling and help them to navigate the pathways to accessing help.

Studies examining perceptions and attitudes of frontline workers have tended not to focus specifically on those working with children and adolescents, except for one study in New Zealand, using focus groups and interviews with 40 frontline youth workers and social service staff to explore their attitudes towards cCBT. This study found some enthusiasm for the approach with cCBT being seen as a potential gateway to getting help (Fleming & Merry, 2013). However, participants also expressed concerns about displacing human contact and about ensuring that it did not cause harm. A survey of 124 mental health workers in Australia found that those with greater knowledge of cCBT also endorsed more advantages and less disadvantages of the approach (Donovan et al., 2015), and a mixed methods study of 11 Swedish primary care therapists found that most thought iCBT should be introduced into their setting, although implementation barriers identified included lack of knowledge and skills, lack of time and resources and unsuitable organisational systems and processes (Kivi et al., 2015); however, neither of these surveys were specific to those working with adolescents.

Given needs-provision gap for adolescents with depression symptoms, it is important to understand how those professionals who work with adolescents but are not mental health specialists view potential solutions like digitally mediated support and treatment. We aimed to investigate frontline professionals’ use of and attitudes towards technology to support adolescents with depression symptoms, including exploring their views on how online self-help SSIs could be used to supplement provision.

## Method

A mixed method, cross-sectional online survey.

### Participants

Anyone working in the UK who self-identified as working in a professional capacity with adolescents, including (but not limited to) education or healthcare professionals, in both public and voluntary sector services, was eligible to participate, providing they were also able to complete measures in English. We particularly targeted recruitment at: General Practitioners (GPs), teachers, school mental health leads, school nurses, Education Mental Health Practitioners (EMHPs) and Child Wellbeing Practitioners (CWPs). The project advert and details were shared via social media (mainly Twitter), university held mailing lists and voluntary sector organisations which support youth mental health, such as Off the Record, Bristol. To incentivize professionals to take part, we offered a prize draw of three £20 shopping vouchers.

**Figure 1.**
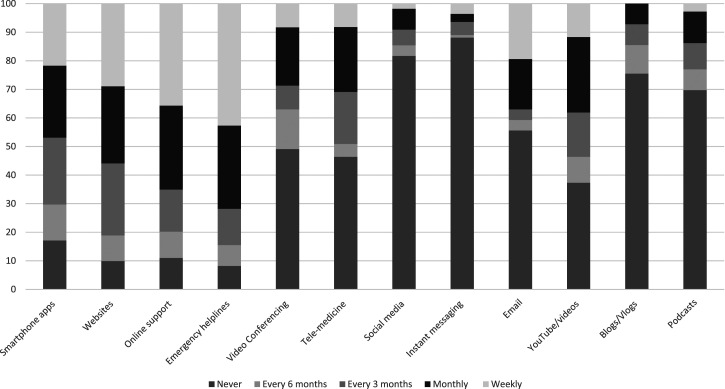
Frequency with which participants are currently using or recommending various media to adolescents struggling with depression symptoms (%).

### Procedure

All study processes took place online via the Qualtrics platform, and the study was open to recruitment from May to July 2023. Prior to taking part in the study, potential participants accessed the study information sheet by clicking on a link/QR code from the study advert. If they wished to proceed, they were invited to complete the online consent form, followed by the study specific measures. EMHP and CWP participants were asked to complete additional measures (via branching logic) for other projects. At the end of the survey, participants were presented with debrief information including links to helpful resources about adolescent depression and information about where participants could seek further support if needed. Responses were anonymous.

Ethical approval was granted by the University of Bath Psychology Research Ethics committee (ref: 23–058). At planning stage, frontline professionals (a GP and an Education Mental Health Practitioner) reviewed the draft study materials and provided feedback and suggestions about how these could be improved. They subsequently offered ideas about how to recruit participants. The study team also included a researcher who had previously worked as a Child Wellbeing Practitioner.

### Measures

Brief demographic information was completed by the participant, including age, gender, geographical area of the UK, and ethnicity. They were then asked to complete some questions about their professional background and experience.

To explore how they currently use/recommend digital mental health interventions to adolescents struggling with their mental health, we used an abbreviated version of Cliffe et al. (2019)’s survey (see supplementary materials S1.). This survey consists of a combination of free text and forced choice responses. Part 1 assesses whether professionals currently use/recommend technology with/to adolescents who are struggling with depression symptoms by asking participants to rate how often they are currently using a range of technologies such as smartphone apps, online CBT and social media: ‘never’, ‘every 6 months’, ‘every 3 months’, ‘monthly’ or ‘weekly’. Part 2 assesses professional’s attitudes towards technology by asking them to indicate their level of agreement to 27 statements: ‘strongly disagree’, ‘disagree’, ‘neither agree/disagree’, ‘agree’, ‘strongly agree’.

We created additional rating scales and free-text boxes to explore what information participants signpost (i.e. direct/make aware of) adolescents with depression symptoms to, exploring barriers and potential solutions, including how online self-help SSIs could be used to supplement provision (see supplementary materials S1. for questions and response options).

### Data analysis

Quantitative data were analysed descriptively (counts, percentages, means). All items were completed by at least 95% of participants, and missingness was <5% per item. Comparison of means was undertaken using pairwise T-tests, with effect sizes (Cohen’s d) interpreted as small (d = .2), medium (d = .5), and large (d = .8) (Cohen, 1988). Bivariate correlations were calculated using Pearson’s, 2 tailed tests.

We used qualitative framework analysis (Gale et al., 2013), combined with principles of reflexive thematic analysis (Braun & Clarke, 2006, 2013; Braun et al., 2020) for more detailed text responses. The steps taken were familiarisation with the data by reading it several times, followed by initial coding and then refinement of codes into themes. We assumed a post-positivist approach, i.e., there is an objective reality, but also acknowledging that people are prone to biases and therefore that knowledge is applicable within the context it is generated. The research team are female psychologists, at different career stages. All have had training in conducting thematic analysis. MEL has extensive experience of working clinically including with adolescents with depression, BC has previously investigated digital mental health interventions and similar topics to the current study albeit in a different group of professionals (i.e., mental health specialists), and GP is a relatively naïve researcher.

## Results

The survey was started 234 times during the data collection period, and of these, 115 (49.1%) participants fully completed the survey and submitted their responses and were retained for analysis. As can be seen in Table 1, most participants were young and cisgender females from a White ethnic group. Most were based in the Southwest region of England, and professional background varied, including staff working in different roles in education, health, and mental health settings.

**Table 1.** Demographic and Professional Characteristics of the Sample (*N* = 115).

Characteristic	Categories	*N*	%
Age (years)	<30	51	44.3
	31–40	29	25.2
	41–50	22	19.1
	51–60	10	8.7
	Not known	3	2.6
Ethnic group	White: English/Welsh/Scottish/Northern Irish/British	96	83.5
	White: (Other)	5	5.2
	Mixed/Multiple ethnic groups	5	5.2
	Asian/Asian British	2	1.7
	Black/African/Caribbean/Black British	3	2.6
Sex (as per official documents)	Male	16	13.9
	Female	96	83.5
Gender	Cisgender	110	95.7
	Transgender	1	0.9
	Not known	4	3.5
Geographical region	England – North East	4	3.5
	England – North West	1	0.9
	England – Yorkshire and the Humber	22	19.1
	England – East Midlands	1	0.9
	England – West Midlands	5	4.3
	England – East of England	5	4.3
	England – London	3	2.6
	England – South East	21	18.3
	England – South West	48	41.7
	Wales	2	1.7
	Not known	3	2.6
Professional background	Clinical/counselling psychologist	5	4.3
	Teacher/teaching assistant/SENCo	20	17.4
	Pastoral staff working in education settings	9	7.8
	School nurse	4	3.5
	GP/GP specialty trainee	8	7
	Counsellor	3	2.6
	Trainee/assistant psychologist	1	0.9
	Child wellbeing practitioner	22	19.1
	Education mental health practitioner	38	33.0
	Other	2	1.7
	Not known	3	2.6
Years working in a professional capacity with adolescents	<1	6	5.2
	1–5	49	42.6
	6–10	25	21.7
	11–15	16	13.9
	16–20	11	9.6
	>20	5	4.3
	Not known	3	2.6

To contextualise the sample, we asked participants to self-rate their technological competence on a 0–10 scale ranging from 2 (intermediate) to 10 (expert). The mean of the sample was 6.41 (SD 1.48). Most participants (*n* = 65, 56.5%) rated themselves as ranging from competent (5) to advanced (7).

Most participants indicated that if they were concerned that an adolescent is struggling with depression symptoms, they would signpost to resources online (*n* = 84, 73%) and arrange a follow-up conversation (*n* = 84, 73%). Just under a third indicated that they would refer the adolescent to mental health services (*n* = 67, 58.3%), and speak to their parents (*n* = 66, 57.4%), and around a half would ask them to fill in a mood questionnaire (*n* = 57, 49.6%). Other responses given in the free text box included undertaking an assessment, providing brief therapeutic support, signposting them to resources within the organisation, and discussing with colleagues.

The most common barriers endorsed to timely help for adolescents struggling with depression were the length of service waiting lists (*n* = 98, 85.2%), and lack of motivation preventing the adolescent from engaging in what is offered (*n* = 78, 67.8%). More than half of the participants indicated that stigma prevents adolescents from engaging in help-seeking (*n* = 65, 56.5%) and that reluctance to tell their parents that they are struggling means adolescents cannot access help (*n* = 60, 52.2%). More than a third endorsed the barriers of services rejecting referrals for help (*n* = 53, 46.1%), it being hard to find an appropriate service to help (*n* = 50, 43.5%) and adolescents not having the resources to travel to service locations (*N* = 39, 33.9%). Less frequently endorsed were the barriers adolescents do not have enough time (*n* = 12, 10.4%), and lack of a confirmed diagnosis means that it is hard to know what to offer (*n* = 24, 20.9%). Other responses given in the free text box included lack of problem recognition by the adolescent or their parents, low mood being more difficult to spot than anxiety, particularly if a young person is ‘quiet but engaging in work’, and lack of early help services. One participant stated that ‘reluctance to engage in virtual support’ could be a barrier.

Figure 1 shows the frequencies with which participants reported using or recommending each of a range of digital media with adolescents struggling with depression symptoms and shows that many frontline professionals recommend online supports like Kooth and emergency helplines like Samaritans fairly regularly, as well as smartphone apps and websites, but social media, instant messaging, blogs and podcasts are much more infrequently used or recommended. When given a free text box and invited to specify resources which they find helpful for adolescents with depression symptoms, participants listed online supports like Kooth and the Mix, digital mental health interventions and apps like Silvercloud, the CalmHarm app and a thinkdiary app, specific YouTube videos, specific websites, (e.g. Young Minds website for adolescents and parents, handouts from the CCI website) and voluntary sector organisations such as Off the Record. They also mentioned resources within their service such as an online wellbeing hub, and specific questionnaires like the goal-based outcomes measure and the low mood subscale of the Revised Children’s Anxiety and Depression Scale (RCADS).

**Figure 2.**
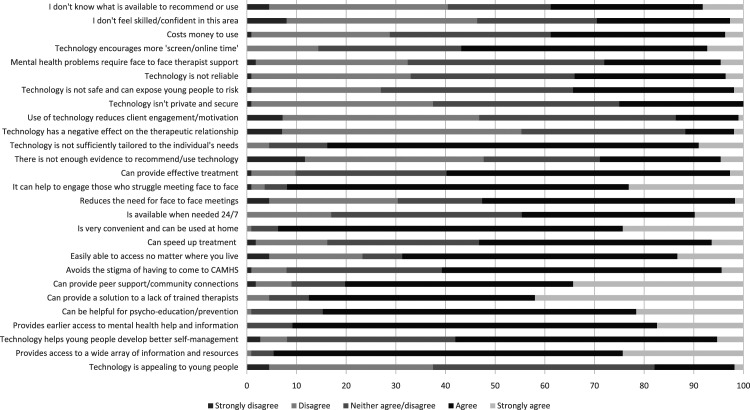
Participant ratings of attitudes towards technology (%).

Overall, participants rated technology as more helpful for adolescents with mild-moderate depression symptoms (mean 6.67, SD 1.70, range 2–10 on a 0–10 scale where 10 = very helpful) as compared to for those with moderate-severe depression symptoms (mean 4.63, SD 1.85, range 1–10), with a large effect size (t(110) = 14.54, *p* < .001, Cohen’s d = 1.49, 95% CI 1.12–1.64). Ratings of perceived helpfulness were not significantly correlated with participant’s self-rated technology competence (r = −.75, *p* = .495 for mild-moderate depression symptoms, r = .05, *p* = .642 for moderate-severe depression symptoms), see Figure 2.

When invited to say what groups of adolescents may be less able to benefit from technology, free text responses included adolescents with comorbidities such as ADHD or autism spectrum conditions, those with learning difficulties/Special Educational Needs, those from low-income households where finances limit availability of devices/Wi-Fi, those who are low in motivation, those whose parents limit access to digital devices, and specific populations e.g. traveller communities, those with limited English language, those who are visually impaired.

### SSIs

**Figure 3.**
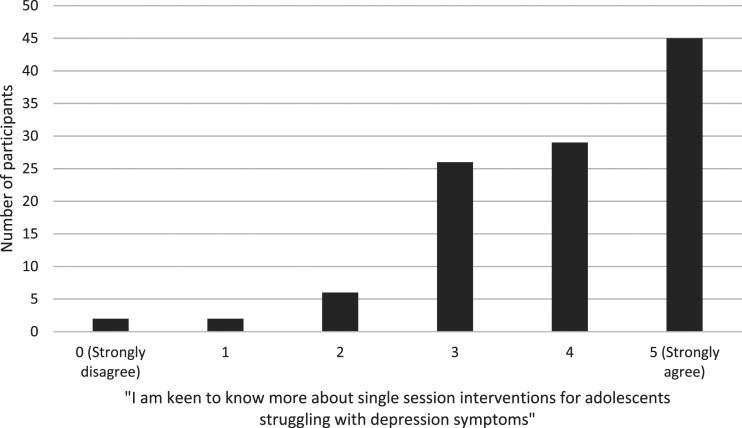
Frequency with which participants agreed that they were interested in knowing more about SSIs.

**Figure 4.**
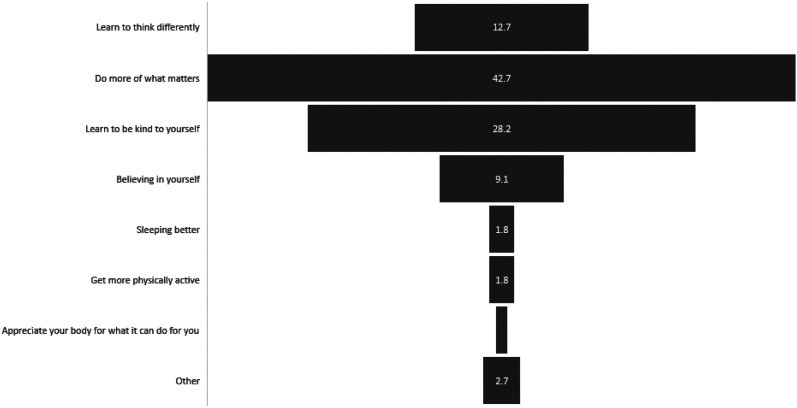
SSI Key Messages rated as most useful and engaging for adolescents with depression symptoms (%).

Most participants were interested in single session interventions (mean = 3.94, SD = 1.15, see Figure 3). Interest levels appeared to be fairly evenly spread across age, gender, and professional background of the participants. Ratings of the potential overall helpfulness of technology for adolescents with mild-moderate symptoms were associated with interest in SSIs (r = .32, *p* < .001), less so for overall helpfulness for moderate-severe symptoms (r = .17, *p* = .077). Interest in SSIs was not significantly associated with self-rated technological competence (r = .06, *p* = .596). The key messages that participants rated as most useful and engaging for adolescents with depression symptoms were ‘Do more of what matters’ (*n* = 47, 40.9%) and ‘Learn to be kind to yourself’ (*n* = 31, 27%), see Figure 4. Additional topics suggested were an SSI focused on online safety and social media risks, and an SSI with a key message ‘You are not alone in your struggle’. Professionals gave ideas in a free text box about how self-help online SSIs could be used for adolescents with depression symptoms, which included as a resource to signpost them to, as a first step intervention, whilst waiting for help, as a part of an overall treatment plan, for those adolescents who are unsure about/unable to commit to engaging in ongoing therapy, and as a ‘top up’ for those who have completed therapy and relapsed. Some suggested that these could be developed into psychoeducational workshops for use in schools, and several mentioned that parents could support the use of the SSIs. One participant stated that SSIs should be ‘only as psychoeducation…but not as a standalone’.

## Discussion

Frontline professionals are adept in using technology and have favourable attitudes towards using this to support adolescents with depression, particularly before the symptoms become too severe. Online resources (e.g., websites) and digital mental health interventions (e.g., apps) are regarded as the most useful and often recommended in the first instance, alongside crisis resources. Professionals also view technology as important in bridging the needs-access gap in traditional mental health services. These might provide early help and a wide range of resources, while being quick and easy to access and negating the stigma associated with using mental health services. However, professionals were concerned that technology may not be useful for all groups and the resources on offer might not be person-centred.

Technology as a source of support was perceived to be more useful for those adolescents with mild-moderate problems, rather than those with more severe problems. This is consistent with findings from surveys of specialist child and adolescent mental health professionals in both Sweden and the UK (Cliffe et al., 2019; Vigerland et al., 2014). This may be due to depression severity tending to be associated with risk and complexity, which may be more difficult to manage safely and in a sufficiently personalised manner using technology. For example, more severe and enduring depression symptoms are associated with self-harm and suicide (Zubrick et al., 2017). In the current study participants typically rated technology as not being tailored to individual’s needs, so it may be that professionals believe that adolescents with more severe depression symptoms may require more individualised support to ensure that high-risk adolescents are kept safe.

The current study used a questionnaire similar to Cliffe et al. (2019) and whilst both sets of findings do align somewhat, there are also some noteworthy differences, particularly considering the pre- and post-pandemic contexts within which the studies took place. The period between Cliffe et al. (2019) and the current study saw the COVID-19 pandemic and consequent rapid adoption of digital technology in mental health services. This may explain why in the current study only 29% agreed that they did not feel skilled in using technology, compared to 42% in Cliffe et al. (2019). Similarly, 38% here agreed that they did not know what technology is available, compared to 60% in Cliffe et al. (2019). Pre-pandemic clinician knowledge of technology was reported as lacking elsewhere, with only around 25% of mental health workers knowing much about CBT in 2015 (Donovan et al., 2015). This may suggest that the accelerated need for and use of technology during the pandemic led to increased awareness and confidence in its use. The current study also found more negative attitudes towards technology, with twice as many participants (83% vs. 42% in Cliffe et al. (2019)) believing that technology is not tailored to adolescents’ needs. Moreover, only 17% believed technology to be appealing to young people, as opposed to 89% in Cliffe et al. (2019). This stark difference may indicate that technology being ‘forced’ upon young people during the pandemic has undermined its appeal, with issues such as digital fatigue being widely reported (e.g., Gregersen et al. (2023); Amponsah et al. (2022)). There is also evidence to suggest that, whilst some young people enjoyed the transition to remote therapy during the pandemic, others found it disruptive and struggled with the online format (Worsley et al., 2022). Lastly, most participants in the current study (87%) believed that technology can be a solution to a lack of trained therapists, compared to only 18% in Cliffe et al. (2019). Unfortunately, the COVID-19 pandemic exacerbated staff shortages within CAMHS, and it may be that the increased familiarity with technology during the pandemic means that practitioners are now more willing to trust the use of technology in CAMHS to ‘fill in the gaps’. It may also be that specialist CAMHS professionals (who were the target population for Cliffe et al. (2019)) have different views to frontline professionals who we recruited in the current study, so we cannot assume that the pandemic is what accounts for the differences between the samples.

It is possible that in the current study, patterns of responses may have differed by the context in which professionals work, for example, rural versus urban location. For example, where travel distances to clinics are greater and public transport options are more limited, professionals may have more favourable views of technology, than in urban centres where travel to clinic locations is likely to be less, with more transport options available. However, we did not collect this information, so future studies are needed to explore this in more detail.

It was encouraging that in our study, frontline professionals were interested in learning about online single session interventions (SSIs) for adolescent depression; this was partly due to the belief that technology might be helpful for supporting adolescents with mild-moderate depression symptoms. Interest in SSIs was unaffected by perceptions that technology might be less useful for more severe depression symptoms and personal technological competence. SSIs were also regarded as useful one-off, preventative, and educational resources for adolescents waiting for treatments and as a follow up for those who have concluded treatment. As described above, staff shortages in mental health services may explain why the majority professionals in the current study believe that technology can help to make up for a lack of trained professionals, contrasting to previous findings (Cliffe et al., 2019). This may also explain the interest in SSIs seen here. Their brief nature also means that SSIs can be developed and adapted to cater to current topics of interest. Their adaptability, diversity and accessibility could mean they can address young people’s needs whilst easing the demand on mental health services.

## Strengths and limitations

We recruited a wide range of non-specialist professionals who work with adolescents, with coverage across different geographical areas of the UK. However, because we used convenience sampling, those who chose to participate who were mainly younger and female, may not necessarily be representative of the wider population of frontline professionals. Furthermore, we relied on self-report measures, and most of our measures were brief to prioritize completion over comprehensiveness. We also used free text boxes to collect explanatory responses rather than in depth interviews, which could be at the expense of detail, and we were not able to probe for further information. When asked about referrals to CAMHS, it is possible that for some participants, this was not an option, depending on service pathways and referral criteria in their local area. There was also high attrition between starting the survey and completing the survey.

## Conclusion

Technology seems to be acceptable to mental health professionals supporting adolescents with depression, particularly adolescents with mild-moderate depression. The increased familiarity with/reliance on technology during the COVID-19 pandemic may have increased clinicians’ understanding of and willingness to trust the use of technology in their practice. There was also interest in the use of SSIs to support adolescents with depression. Technology, including SSIs may be particularly helpful for prevention, early intervention or supporting those on waiting lists. Future research should seek to further evaluate the use of SSIs for adolescent depression.

## Supplemental Material

Supplemental Material - Frontline professionals’ use of and attitudes towards technology to support interventions for adolescents with depression symptoms: A mixed methods survey Supplemental Material for Frontline professionals’ use of and attitudes towards technology to support interventions for adolescents with depression symptoms: A mixed methods survey by ME Loades, B Cliffe and G Perry in Clinical Child Psychology and Psychiatry.
