# Supplementary material for: Frontline professionals’ use of and attitudes towards technology to support interventions for adolescents with depression symptoms: A mixed methods survey
Source: Clin Child Psychol Psychiatry. 2023 Nov 6;29(3):1087–99. doi: 10.1177/13591045231212523 (PMC11188557; doi:10.1177/13591045231212523)
Supplement: Supplemental Material - Frontline professionals’ use of and attitudes towards technology to support interventions for adolescents with depression symptoms: A mixed methods survey [file sj-pdf-1-ccp-10.1177_13591045231212523.pdf]

S1. QUESTIONS POSED TO RESPONDENTS

- What do you do if you are concerned that an adolescent is struggling with depression symptoms (e.g. low mood/irritability, lack of enjoyment, sleep problems, fatigue)? (please select all that apply)
    - o Signpost to resources online
    - o Arrange a follow-up conversation
    - o Refer to mental health services
    - o Ask them to fill in a mood questionnaire
    - o Speak to their parents
    - o Other (please specify)

- What gets in the way of getting *timely* help for adolescents who are struggling with depression? (please select all that apply)
    - o Stigma prevents the adolescent from engaging in help-seeking
    - o It is hard to find an appropriate service to help.
    - o Services reject referrals for help.
    - o Services have long waiting lists.
    - o Adolescents do not have enough time.
    - o Adolescents do not have the resources to travel to service locations.
    - o Lack of motivation prevents the adolescent from engaging in what is offered.
    - o Lack of a confirmed diagnosis means that it is hard to know what to offer.
    - o Reluctance to tell their parents that they are struggling means adolescents cannot access help.
    - o Other (please specify)

Abbreviated version of Cliffe et al. (2019) – Attitudes towards technology

- Please use the scale below to select which number best describes how you would describe your technological competence

Novice *Intermediate* *Competent* *Advanced* *Expert*

○ ○ ○ ○ ○ ○ ○ ○ ○ ○ ○

0 1 2 3 4 5 6 7 8 9 10

- How often are you **currently using or recommending** the following to adolescents struggling with depression symptoms?

Never Every 6 Every 3 Monthly Weekly

		**months**	**months**		
**Smartphone apps** (e.g. BlueIce, Calm Harm)					
**Websites** (e.g. Headspace)					
**Online support** (e.g. Kooth)					
**Emergency Helplines** (e.g. Samaritans, Childline)					
**Video conferencing** (e.g. Zoom)					
**Tele-medicine** (e.g. telephone follow-ups)					
**Social media** (e.g. Instagram, Twitter, TikTok)					
**Instant messaging** (e.g. Snapchat)					
**Email**					
**YouTube/videos** (e.g. TED talks)					
**Blogs/Vlogs** (e.g. mental elf, purple persuasion)					
**Podcasts**					

Please list any specific resources that you are currently using and finding helpful for adolescents with depression symptoms.

- To what extent do you agree or disagree with the following statements

	Strongly disagree	Disagree	Neither agree/disagree	Agree	Strongly agree
There is not enough evidence to recommend/use technology	○	○	○	○	○
I don't feel skilled/confident in this area	○	○	○	○	○
It can help to engage those who struggle meeting face to face	○	○	○	○	○
I don't know what is available to recommend or use	○	○	○	○	○
Technology is appealing to young people	○	○	○	○	○
Is available when needed 24/7	○	○	○	○	○
Technology is not sufficiently tailored to the individual's needs	○	○	○	○	○
Can provide a solution to a lack of trained therapists	○	○	○	○	○
Can provide peer support /community connections	○	○	○	○	○
Technology has a negative effect on the therapeutic relationship	○	○	○	○	○
Use of technology reduces client engagement/motivation	○	○	○	○	○
Avoids the stigma of having to come to CAMHS	○	○	○	○	○
Technology isn't private and secure	○	○	○	○	○
Technology is not safe and can expose young people to risk	○	○	○	○	○
Can provide effective treatment	○	○	○	○	○

Technology helps young people develop better self-management	○	○	○	○	○
Reduces the need for face-to-face meetings	○	○	○	○	○
Technology is not reliable	○	○	○	○	○
Easily able to access no matter where you live	○	○	○	○	○
Provides access to a wide array of information and resources	○	○	○	○	○
Mental health problems require face to face therapist support	○	○	○	○	○
Can be helpful for psychoeducation/ prevention	○	○	○	○	○
Technology encourages more 'screen/online time'	○	○	○	○	○
Costs money to use	○	○	○	○	○
Provides earlier access to mental health help and information	○	○	○	○	○
Can speed up treatment	○	○	○	○	○
Is very convenient and can be used at home	○	○	○	○	○

Overall, **how helpful** do you think technology can be for adolescents with *mild-moderate* depression symptoms?

Not helpful *Very helpful*

○	○	○	○	○	○	○	○	○	○	○
0	1	2	3	4	5	6	7	8	9	10

Overall, **how helpful** do you think technology can be for adolescents with *moderate-severe* depression symptoms?

Not helpful *Very helpful*

○ ○ ○ ○ ○ ○ ○ ○ ○ ○ ○

0 1 2 3 4 5 6 7 8 9 10

- What groups of adolescents do you think are less able to use and benefit from technology?

Online SSIs

An single session intervention is a brief, one-time intervention that is designed to provide individuals with immediate support and guidance for a specific issue or problem. This type of intervention is often delivered as 'self-help' online, and can be accessed at any time and from any location. It may include written and/or audio-visual materials, interactive exercises, and links to additional resources.

- I am keen to know more about single session interventions for adolescents who are struggling with depression symptoms.

Strongly disagree *Strongly agree*

○ ○ ○ ○ ○ ○

0 1 2 3 4 5

Online, self-help single session interventions They can be used as standalone interventions or as a supplement to other forms of treatment, such as therapy or medication. If you were given information about online, self-help SSIs, designed for and with adolescents, that were shown to reduce depression symptoms, how would you use these?

- Each SSI has a key message. The key message I think would be most useful and engaging for adolescents with depression symptoms are:
    - Learn to think differently
    - Do more of what matters
    - Learn to be kind to yourself
    - Believing in yourself
    - Sleeping better
    - Get more physically active
    - Appreciate your body for what it can do for you
    - Other (please suggest any topics you think would be useful for teens)

References

Cliffe, B., Croker, A., Denne, M., & Stallard, P. (2019). Clinicians' use of and attitudes towards technology to provide and support interventions in child and adolescent mental health services. *Child Adolesc Ment Health*. https://doi.org/10.1111/camh.12362

S2. Table 2. Frequency with which participants are currently using or recommending various media to adolescents struggling with depression symptoms – n (%)

	Smartphone apps e.g. BlueIce, Calm Harm	Websites (e.g. Headspace)	Online support (e.g. Kooth)	Emergency helplines (e.g. Samaritans, Childline)	Video Conferencing (e.g. Zoom)	Tele-medicine (e.g. telephone follow-ups)	Social media (e.g. Instagram, Twitter, TikTok)	Instant messaging (e.g. Snapchat)	Email	YouTube/videos (e.g. TED talks)	Blogs/Vlogs (e.g. Mental Elf, purple persuasion	Podcasts
**Never**	19 (17.1)	11 (9.9)	12 (11.0)	9 (8.2)	53 (49.1)	51 (46.4)	89 (81.7)	96 (88.1)	60 (55.6)	41 (37.3)	83 (75.5)	76 (69.7)
**Every 6 months**	14 (12.6)	10 (9.0)	10 (9.2)	8 (7.3)	15 (13.9)	5 (4.5)	4 (3.7)	1 (0.9)	4 (3.7)	10 (9.1)	11 (10.0)	8 (7.3)
**Every 3 months**	26 (23.4)	28 (25.2)	16 (14.7)	14 (12.7)	9 (8.3)	20 (18.2)	6 (5.5)	5 (4.6)	4 (3.7)	17 (15.5)	8 (7.3)	10 (9.2)
**Monthly**	28 (25.2)	30 (27.0)	32 (29.4)	32 (29.1)	22 (20.4)	25 (22.7)	8 (7.3)	3 (2.8)	19 (17.6)	29 (26.4)	8 (7.3)	12 (11.0)
**Weekly**	24 (21.6)	32 (28.8)	39 (35.8)	47 (42.7)	9 (8.3)	9 (8.2)	2 (1.8)	4 (3.7)	21 (19.4)	13 (11.8)	0	3 (2.8)

Note: all items were optional and not every participant responded to every item; %s are of those who responded to that item
